# NeRvolver: a computational intelligence-based system for automated construction, tuning, and analysis of neuronal models

**DOI:** 10.1186/1471-2202-13-S1-P36

**Published:** 2012-07-16

**Authors:** Emlyne Forren, Myles Johnson-Gray, Parth Patel, Tomasz G Smolinski

**Affiliations:** 1 Department of Computer and Information Sciences, Delaware State University, Dover, DE 19901, USA

## 

Building neuronal models, even on the single-cell level, requires tremendous amounts of effort invested in the design of a realistic and functioning structure (*e.g.*, the number and characteristics of compartments) and proper tuning of the model’s parameter values (*e.g.*, membrane and axial conductances). Due to the ever-increasing computational power of modern computers facilitating the use of more and more complex neuronal models, hand-tuning of models’ parameters is becoming less and less feasible. Therefore, automated methods for neuronal model construction have been lately gaining much attention. Although a significant number of techniques for automatic model parameter estimation have been recently proposed (*e.g.*, [1,3,4]), they are predominantly limited to “fine-tuning” of already designed models in a predetermined search space of parameter values. Furthermore, although unquestionably successful, present approaches are almost exclusively domain-specific, designed to deal with particular neuron types, or explicit classes of electrical activity (*e.g.*, spiking neurons). Finally, most of the existing systems attempt to produce a single, best-matching model, and rely on a direct comparison between the voltage trace of the model and its biological equivalent.

Here, we propose a computational intelligence-based system for automated construction and tuning of neuronal models that builds upon the existing research in the area of automatic neuron model parameter optimization, but at the same time significantly expands the current approaches. The system, called NeRvolver, is designed to not only optimize the parameters of existing neuronal models, but also to allow for creation of entire sets of models matching some predefined criteria (*e.g.*, in terms of the neuron’s characteristics such as spike height, inter-spiking interval, burst duration, period, *etc*.) “from scratch” (by utilizing, for example, only a limited set of generic currents as the starting point in the process of model construction). Furthermore, in addition to generating neuronal models, through the hybridization of multi-objective evolutionary algorithms (MOEA) and fuzzy logic (FL), the system generates classification rules describing biological phenomena discovered during the process of model creation or tuning (*e.g.*, “**IF** sodium axon conductance is low, **THEN** spike frequency is low”). The purpose of generating such rules is twofold: 1) they can be used in subsequent runs of the modeling algorithm to improve its convergence time, and 2) they may potentially provide insights into the functioning of the biological neurons being modeled. For example, by using the aforementioned rule, the system may automatically increase the sodium axon conductance across the individuals in the evolutionary algorithm’s current population, if a higher spike frequency is desired. This is akin to the idea of local improvement of the genetic material using memes in memetic algorithms [2]. In addition, based on the above-quoted rule, the conclusion may be drawn that sufficient sodium axon conductance is necessary for adequate spike frequency. Such inferences, here automatically generated by the modeling system, can obviously be extremely useful for the understanding of the underlying biological phenomena.

## References

[B1] Druckmann S Banitt Y Gideon A Schurmann F Markram H Segev I A novel multiple objective optimization framework for automated constraining of conductance-based neuron models by noisy experimental data Front Neurosci 2007 1 7 18 10.3389/neuro.01.1.1.001.2007 18982116 PMC2570085

[B2] Moscato P On Evolution, Search, Optimization, Genetic Algorithms and Martial Arts: Towards Memetic Algorithms Caltech Concurrent Computation Program 1989 Report 826

[B3] Prinz AA Billimoria CP Marder E Alternative to hand-tuning conductance-based models: Construction and analysis of databases of model neurons J Neurophysiol 2003 90 3998 4015 10.1152/jn.00641.2003 12944532

[B4] Van Geit W Achard P De Schutter E Neurofitter: A parameter tuning package for a wide range of electrophysiological neuron models Frontiers in Neuroinformatics 2007 1 1 18 18974796 10.3389/neuro.11.001.2007 PMC2525995

